# Differences of anticholinergic drug burden between older hospitalized patients with and without delirium: a systematic review and meta-analysis based on prospective cohort studies

**DOI:** 10.1186/s12877-024-05197-6

**Published:** 2024-07-12

**Authors:** Chifong Ieong, Tingjia Chen, Sai Chen, Xiang Gao, Kemin Yan, Wen He, Hua Hong, Yong Gu, Xiao Chen, Gang Yuan

**Affiliations:** 1 Department of Geriatrics, The First Affiliated Hospital of Sun Yat-sen University, Guangzhou, China; 2 Department of Pharmacy, The First Affiliated Hospital of Sun Yat-sen University, Guangzhou, China; 3 Healthcare Center, The First Affiliated Hospital of Sun Yat-sen University, Guangzhou, China

**Keywords:** Anticholinergic drug burden, Older hospitalized patients, Relationship, Delirium, Meta-analysis

## Abstract

**Objectives:**

This review aims to comprehensively summarize the differences in anticholinergic drug burden (ADB) scores between older hospitalized patients with and without delirium.

**Methods:**

We searched PubMed, Embase, Web of Science, Cochrane Library and CINAHL EBSCOhost databases to identify prospective cohort studies exploring the relationship between ADB and the occurrence of delirium in older hospitalized patients. The primary outcome of the review was the mean ADB scores for the delirium and non-delirium groups, and the secondary outcome was the scores for the subsyndromal and non-delirium groups. The standardized mean difference (SMD) and corresponding 95% confidence intervals (95% CI) were incorporated using a fixed-effect method. Moreover, we performed subgroup analysis according to the admission type, age, the ADB scale type and the ADB classification.

**Results:**

Nine prospective cohort studies involving 3791 older patients with a median age of 75.1 (71.6–83.9) were included. The ADB score was significantly higher in the delirium group than in the non-delirium group (SMD = 0.21, 95%CI 0.13–0.28). In subgroup analysis, the age subgroup was split into < 75 and ≥ 75 according to the median age of the older people. There were significant differences in ADB scores between older people with delirium and those without delirium in various subgroups: surgical (SMD = 0.20, 95%CI 0.12–0.28), internal medicine (SMD = 0.64, 95%CI 0.25–1.02), age < 75 (SMD = 0.17, 95%CI 0.08–0.26), age ≥ 75 (SMD = 0.27, 95%CI 0.15–0.39), ADS scale (SMD = 0.13, 95%CI 0.13–0.40), ARS scale (SMD = 0.15, 95%CI 0.03–0.26), ACB scale (SMD = 0.13, 95%CI 0.01–0.25), pre-admission ADB (SMD = 0.24, 95%CI 0.05–0.43) and ADB during hospitalization (SMD = 0.20, 95%CI 0.12–0.27).

**Conclusions:**

We found a quantitative relationship between ADB and delirium in older patients admitted for internal medicine and surgery. And this relationship remained significant in different age, ADB scale type and ADB classification subgroups. However, the actual difference in ADB scores between patients with delirium and without delirium was small. More high-quality observational studies should be conducted to explore the impact of ADB on delirium and subsyndromal delirium.

**Clinical Trial Registration:**

The protocol was published in the International Prospective Register of Systematic Reviews (PROSPERO) [Ref: CRD42022353649].

**Supplementary Information:**

The online version contains supplementary material available at 10.1186/s12877-024-05197-6.

## Introduction

To date, central cholinergic deficiency is the leading hypothesized mechanism for delirium [1]. Acetylcholine plays an important role in maintaining attention, memory and consciousness [2]. Any process that interferes with the physiological effect of acetylcholine may cause core delirium symptoms such as inattention, altered level of consciousness, disorientation, and perceptual disturbances [3]. Drugs with anticholinergic effects are associated with delirium, which affect acetylcholine by antagonizing postsynaptic muscarinic receptors or other mechanisms [3]. Ten of 25 drugs commonly used in the elderly for the treatment of allergic reactions, depression, Parkinson’s disease, dizziness, asthma, psychiatric symptoms, and behavioral problems, have been reported to have anticholinergic effects [4]. The older people are more vulnerable to anticholinergic adverse effects due to comorbidities, age-related changes in physiological functions, pharmacokinetics and pharmacodynamics [5]. According to the AGS Beers Criteria 2019, the use of potent anticholinergic drugs is a risk factor for delirium in the older people [6].

Anticholinergic drug burden (ADB) reflects the cumulative anticholinergic effect of drugs [7]. A variety of simple and quantitative scoring scales have been developed for ADB based on the anticholinergic intensity of the drug. The total ADB can be determined by summing up the ADB scores of each drug.

Several studies have shown the impact of ADB scores on delirium, but the results are controversial due to study design, population, and sample size [8–10]. Previous systematic reviews have summarized the correlation between ADB scores and delirium and they compared the associations between different ADB scales and delirium with the aim of identifying the most relevant ADB scale to identify individuals at high risk of delirium [11, 12]. However, these reviews only reported effect sizes for each study without meta-analysis and could not provide a quantitative relationship between ADB and delirium, particularly in older hospitalized individuals.

Our objective is to conduct a systematic review and meta-analysis of prospective cohort studies to investigate and quantify the effect of ADB on delirium in older hospitalized patients. This would be beneficial in preventing delirium and managing the use of anticholinergic drugs in older hospitalized patients.

## Methods

This systematic review and meta-analysis was registered on PROSPERO (CRD42022353649) and conducted in accordance with the Preferred Reporting Items for Systematic Reviews and Meta-Analysis (PRISMA) guidelines. The accompanying checklist can be found in Supplementary Table S1.

### Data sources and search strategy

A systematic literature search was conducted using PubMed, Embase, Web of Science, Cochrane Library and CINAHL EBSCOhost databases, covering the period up to December 26, 2022, with no language restrictions. The following search terms were used: aged, cholinergic antagonist, and delirium. Boolean operators were used to combine different search terms. The specific search strategy for each database is outlined in Supplementary Table S2.

### Eligibility criteria

Inclusion criteria: (1) Prospective cohort studies exploring the association between ADB and the occurrence of delirium in hospitalized patients aged ≥ 65 years. (2) ADB as measured by the anticholinergic rating scales. Exclusion criteria: (1) No mean/median ADB or the OR value based on the continuous ADB. (2) No diagnostic criteria for delirium were available. (3) No non-delirium control group was provided. (4) Patients with cognitive impairment such as Alzheimer’s disease. (4) No information available for meta-analysis.

### Study screening and selection

All literature search results were imported into Endnote X9 (Clarivate Analytics, Philadelphia, PA) for initial screening, and duplicate articles were removed. Irrelevant articles were excluded based on title and abstract, while those that met the eligibility criteria were identified through full-test reading. Two reviewers (CI and TC) independently performed the screening and selection of studies, while final included articles were cross-checked. If two reviewers disagreed on a controversial article, a third reviewer (GY) should be consulted. All three reviewers must discuss and agree on the final decision before including an article in the review.

### Data extraction

Two reviewers (CI and TC) independently used a pre-designed table to extract the following information from the articles: (1) study characteristics (authors, year of publication, study design, setting, language, country); (2) participant characteristics (type of admission, study population, sample size, age and sex of participants); (3) ADB (ADB measurements, type of ADB); (4) delirium (incidence/prevalence of delirium, assessment methods for delirium); (5) information from statistical analyses (number of patients in delirium and non-delirium groups, mean ADB scores and corresponding standard errors for both groups, odds ratios and corresponding 95% confidence intervals reflecting the relationship between ADB and delirium). Any disagreements were discussed by the three reviewers.

### Quality assessment

CI and TC independently assessed the quality of the articles using the Newcastle Ottawa Scale, which was designed for cohort and case-control studies [13]. Each article was evaluated on eight items and categorized into three groups: Selection of Study Groups (score range 0–4); Comparability of Groups (score range 0–2) and Determination of Exposure/Outcome (score range 0–3). The total score ranged from 7 to 9 for high quality, 4 to 6 for low quality, and 0 to 3 for very low quality. Any disagreements were discussed by the three reviewers.

### Statistical analysis

The primary outcome of our review was the mean ADB score for the delirium and non-delirium groups, while the scores for subsyndromal delirium and non-delirium groups were considered as secondary outcomes. The median ADBs in the studies were converted to mean ADBs according to the Cochrane Handbook [14]. Since the mean ADB score is a continuous variable, standardized mean difference (SMD) and standard errors (SE) were used as effect sizes. If the studies reported the OR values based on the continuous ADB scores with the corresponding SE, we calculated the effect size using the following formula: $\:\text{SMD=}\frac{\sqrt{\text{3}}}{\text{n}}\text{lnOR}$ [14]. The SE of the log OR can be converted to the SE of the SMD by multiplying by the same constant ($\:\frac{\sqrt{\text{3}}}{\text{n}}$) [14]. The inverse-variance fixed‐effect method was used to calculate the pooled effect sizes (SMD) with SE. According to the interpretation of effect size in Cohen’s guideline: effect size = 0.2 is considered a “small” effect size, 0.5 represents a “medium” effect size, and 0.8 a “large” effect size [15]. All *p*-values ​​were two-sided, and 0.05 was considered to be statistically significant. Statistical heterogeneity was assessed by the *I*^2^ test and Galbraith plot, with *p* < 0.10 for the *I*^2^ test indicating significant heterogeneity. Publication bias was tested by funnel plot and Egger’s test. All analyses were conducted using Stata (version 16.0, StataCorp, College Station, Texas).

### Subgroup analysis and sensitivity analysis

We performed subgroup analyses based on the following variables: Type of admission (surgical, internal medicine, emergency), age, type of ADB scales and the classification of ADB (pre-admission or during hospitalization). The statistical method used in the subgroup analysis are consistent with those used in the overall analysis. The inverse-variance fixed‐effect method was used to calculate the pooled effect sizes (SMD) with SE. For sensitivity analyses, we used the leave-one-out sensitivity test to evaluate the effect of individual studies on the results and the robustness. Meta-analysis was again performed, removing each study in turn to obtain a new subset of studies, resulting in a new pooled SMD that compared with the original pooled SMD.

## Results

### Study selection

**Fig. 1:**
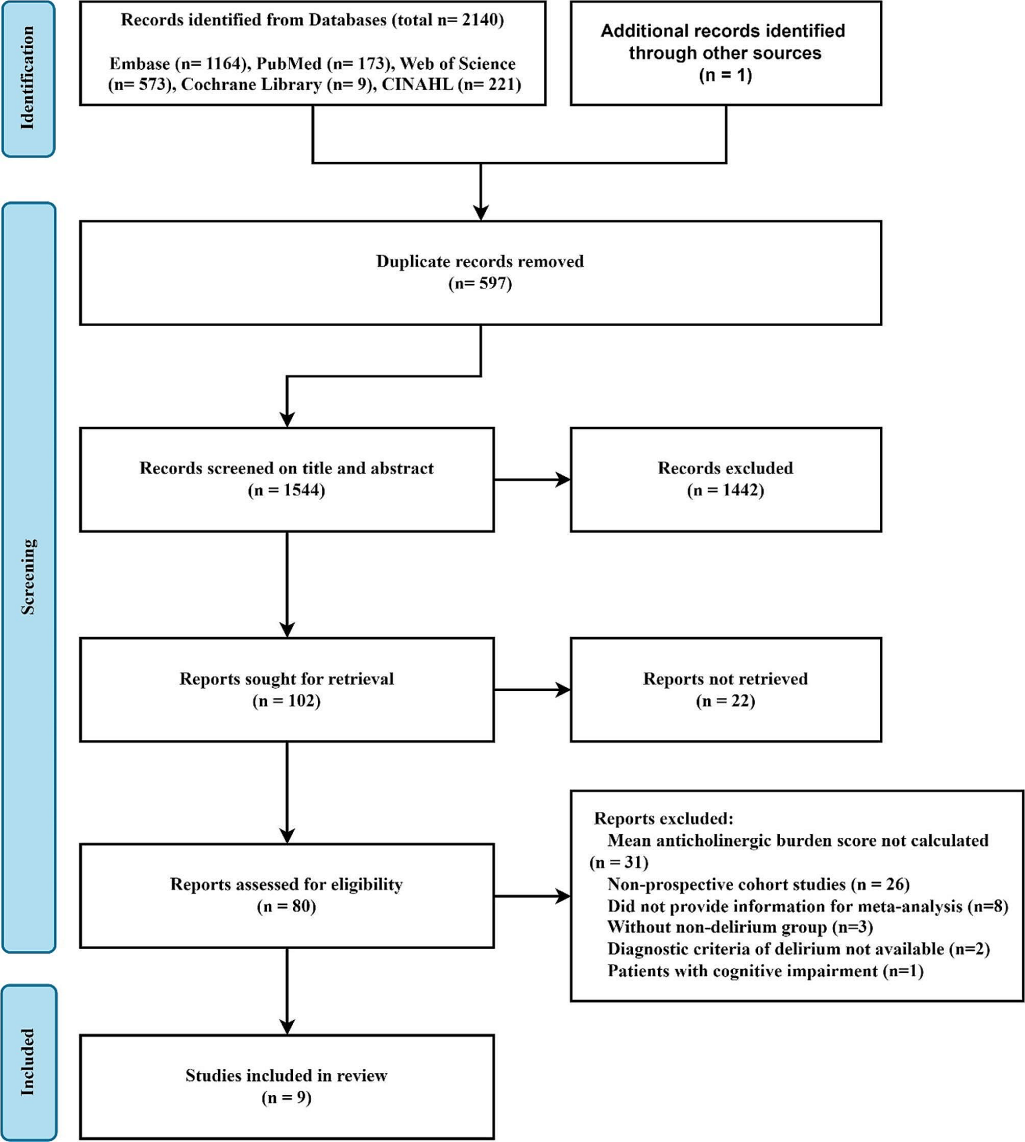
PRISMA flow diagram of the study selection process

We retrieved 2141 articles with our search strategy. After removing 597 duplicates and 1442 irrelevant articles based on their titles and abstracts, a total of 102 were assessed for eligibility. Finally, 9 articles of 80 available full-text articles were included in this systematic review and meta-analysis. The flow chart of article screening is shown in Fig. 1.

### Study characteristics

A total of nine prospective cohort studies were obtained (Table 1), including 3791 older hospitalized patients with a median age of 75.1 (71.6–83.9) [16–22]. The median incidence of delirium was 28.3% (5.1-51%), and the incidence of subsyndromal delirium ranged from 14.6 to 17%. All articles were written in English, with seven studies conducted in Europe [16, 18–20, 22, 23], one in Asia [17], and one in the United States [11]. Patients in five studies were admitted for surgery [16, 18, 20, 22, 23], two for internal medicine treatment [12, 17], and two for emergency department visits [11, 19]. ADB was measured using the Anticholinergic Cognitive Burden scale (ACB) [16–19], the Anticholinergic Drug Scale (ADS) [16, 18, 20, 23], the Anticholinergic Risk Scale (ARS) [18, 22] and the German anticholinergic burden score (GABS) [17]. Four studies calculated pre-admission ADB [17, 19, 20], five studies calculated ADB during hospitalization [11, 16, 18, 22, 23]. Four studies reported the OR value as the statistical indicator of the outcome [11, 12, 22, 23], and five studies directly reported ADB scores in the delirium and non-delirium groups [16–20]. Delirium was assessed using various tools such as Confusion Assessment Method (CAM) [16–18, 20, 22], short-form CAM [12, 19], CAM for ICU (CAM-ICU) [11, 18, 23], modified CAM [11], Nursing Delirium Scale (Nu-DESC) [18, 23], and Delirium Observation Screening Scale (DOS) [20]. In addition to delirium, two of the nine studies further explored the impact of ADB on subsyndromal delirium [17, 19]. Subsyndromal delirium is a transitional state between normal consciousness and delirium, with some abnormal features in delirium assessment, but does not meet all criteria for delirium diagnosis [24].

**Table 1:** Study characteristics

Author	Language (country)	Study population	Sample size, *n*	Age (mean, SD)	Sex male (*n*, %)	Delirium incidence	Delirium prevalence	ADB type	ADB scale (calculation)	Outcome	Delirium assessment	Results ADB score (mean, SD) or OR (95% CI)
Cerejeira [16], 2011	English (Portugal)	Patients undergoing elective total hip replacement	101	73.04 (6.29)	50 (49.5)	36.6%		During hospitalization	ADS (sum)	Postoperative delirium	CAM	Delirium: 0.84 (1.32) Non-delirium: 0.59 (0.69)
Efraim [17], 2020	English (Israel)	patients aged over 65 years hospitalized in the Division of Internal Medicine	158	75.3 (7.4)	87 (55.1)		5.1% (full delirium) 14.6% (subsyndromal delirium)	Pre-admission	GABS (sum)	Delirium, subsyndromal delirium during hospitaliazation	CAM	Full delirium: 3.5 (1.83) Subsyndromal delirium: 2.7 (1.5) Non-delirium: 1.6 (1.5)
Heinrich [18], 2021	English (Germany and the Netherlands)	Patients aged over 65 years undergoing elective surgery	837	71.59 (5.31)	475 (56.6)	19.7%		During hospitalization	ADS ACB ARS (sum)	Postoperative delirium	DSM-5; CAM; CAM-ICU; Nu-DESC	Delirium: ADS 0.45 (0.92); ARS 0.12 (0.5); ACB 0.53 (0.97) Non-delirium: ADS 0.39 (0.86); ARS 0.13 (0.54); ACB 0.39 (0.77)
Mueller [21], 2020	English (Germany)	Patients aged 65 years and older scheduled for surgery for gastrointestinal, genitourinary, gynecological or thoracic cancers	651	71.8 (4.9)	446 (68.5)	10.1%		During hospitalization	ADS (sum)	Postoperative delirium	CAM-ICU; Nu-DESC	Per point: 1.496 (1.09–2.052) > 0 vs. 0: 2.215 (1.214–4.039)
Noel [11], 2019	English (USA)	Emergency Department patients who were 65 years and older	228	73.92 (9.66)	102 (44.7)		46.1%	During hospitalization	ACB (sum)	Delirium at admission	Modified CAM; CAM-ICU	Per point: 0.7 (0.4 ~ 1.2)
Rawle [19], 2021	English (United Kingdom)	Patients aged over 70 years with an emergency unplanned admission	577	83.2 (7.4)	237 (41)		13.3% (full delirium) 17.0% (subsyndromal delirium)	During hospitalization	ACB (sum)	Delirium and subsyndromal delirium during hospitalization	Short-form CAM	Full delirium: 1.2 (1.6) Subsyndromal delirium: 1.2 (1.4) Non-delirium: 1.1 (1.4)
Rigor [12], 2020	English (Portugal)	Patients 65 years of age and older who were admitted to an Internal Medicine ward	198	79.9 (7.5)	106 (53.5)		28.3%	During hospitalization	ACB (sum)	Delirium during hospitalization	Short-form CAM	Per point: 1.65 (1.09 ~ 2.51)
Van Munster [20], 2012	English (the Netherlands)	Patients aged 65 years and older admitted with acute hip fracture and scheduled for operation	142	83.87 (6.96)	42 (29.6)	51.0%		Pre-admission	ADS (sum)	Postoperative delirium	CAM; DOS	Delirium: 0 (0.74) Non-delirium: 0 (0.74)
Herrmann [22], 2022	English (Germany)	Patients aged 70 years and older scheduled for elective surgery with an expected duration of surgery of at least 60 min.	899	77.3 (4.9)	455 (50.6)	23.4%		Pre-admission	ARS (sum)	Postoperative delirium in the first 7 days	CAM; DSM-5	Per point: 1.54 (1.15–2.02)

### Quality of the studies

The results of the quality assessment according to the NOS are presented in Supplementary Table S3. The overall quality of all nine studies was high, with an average score of 8.3 (range from 7 to 9). Seven studies scored 4 in the Selection Group and six studies scored 2 in the Comparability Group. All studies scored 3 points in the Outcome Group.

### Primary outcome and subgroup analysis

**Fig. 2:**
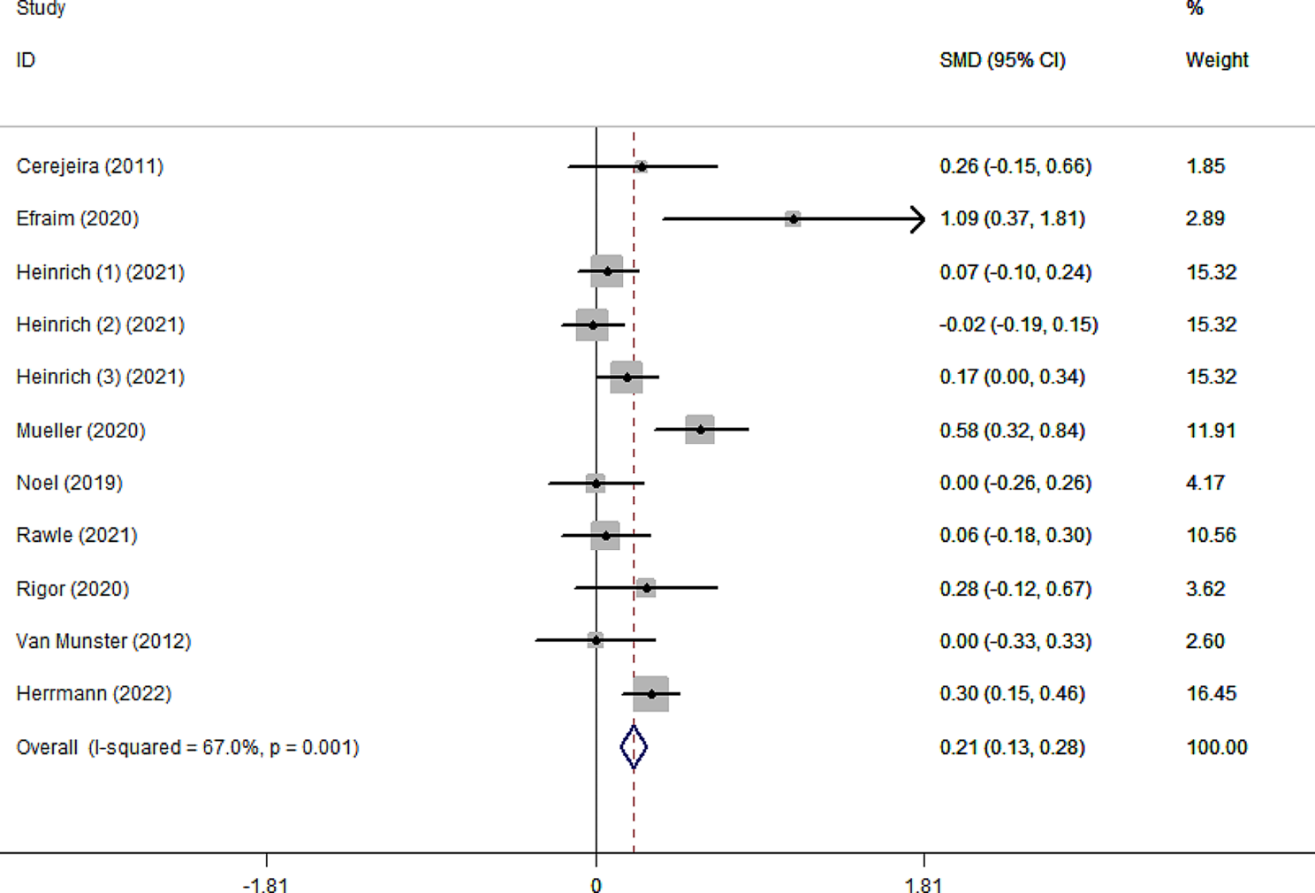
Forest plot for the effects of ADB score on delirium. The black solid diamond and horizontal lines represent the corresponding SMD and 95% CIs in each study, while the vertical red dotted line suggests the corresponding pooled SMD. The gray boxes point to the weight for each study. The blue diamond corresponds to the overall SMD and 95% CI

**Fig. 3:**
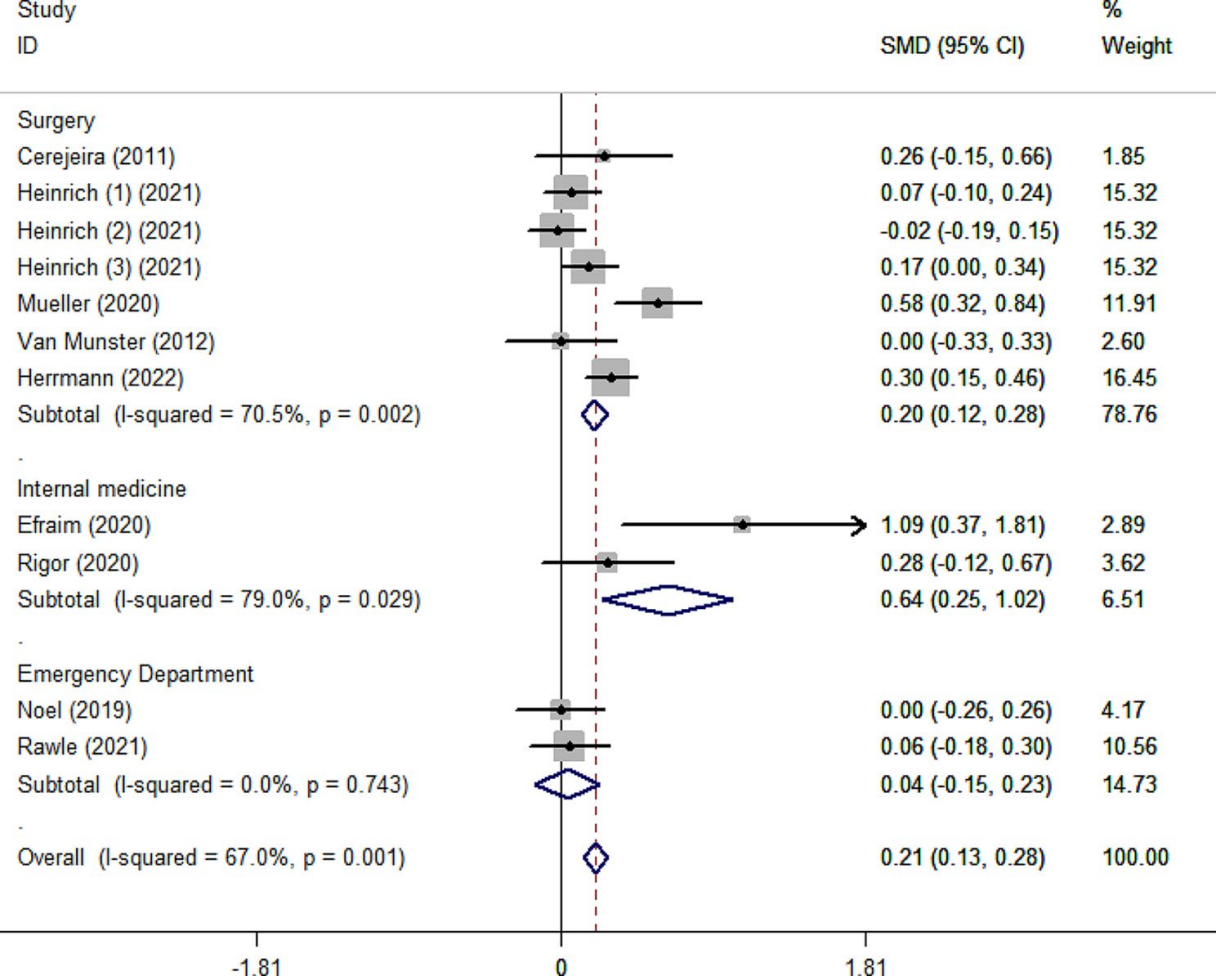
Forest plot for subgroup analysis according to the admission type. The black solid diamond and horizontal lines represent the corresponding SMD and 95% CIs in each study, while the vertical red dotted line suggests the corresponding pooled SMD. The gray boxes point to the weight for each study. The blue diamond corresponds to the overall SMD and 95% CI

**Fig. 4:**
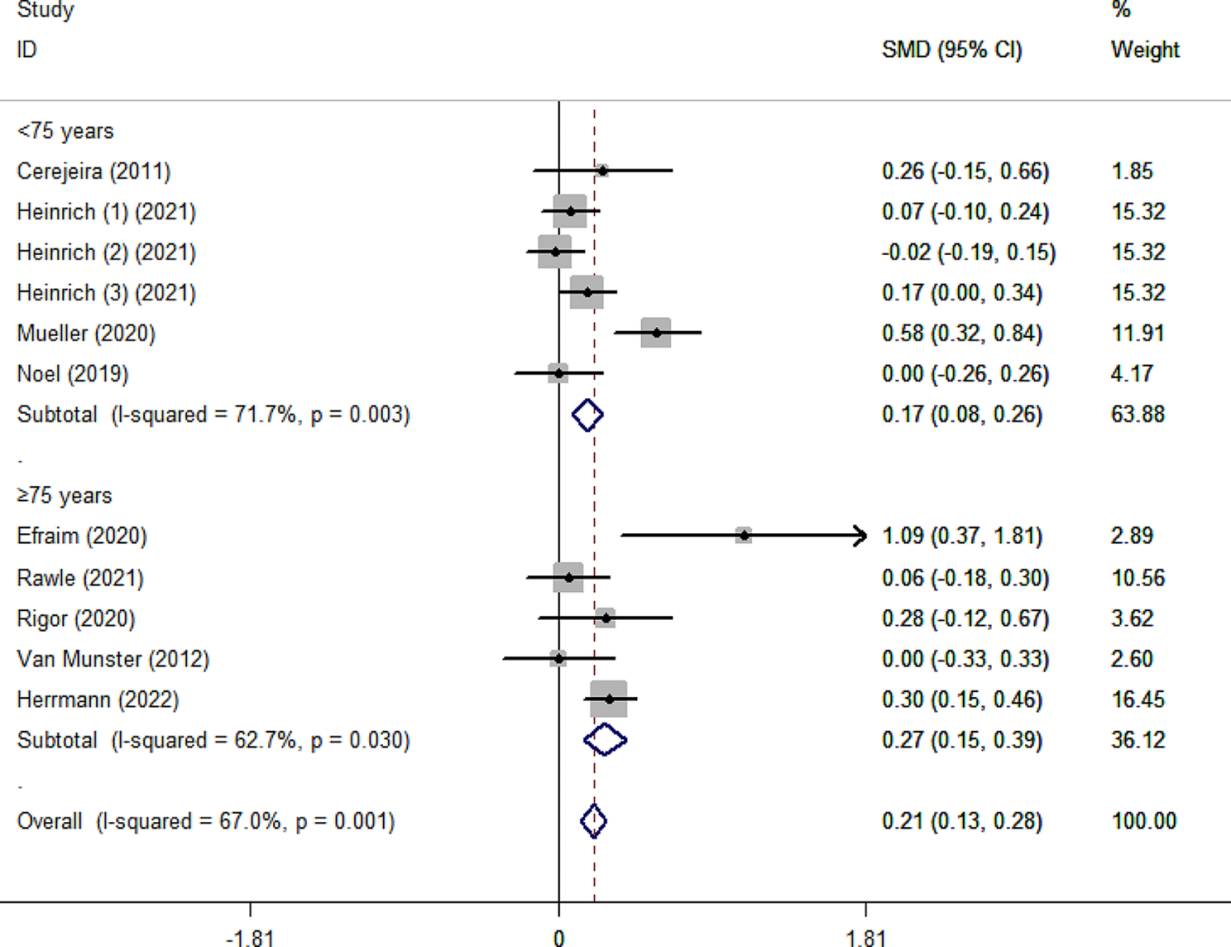
Forest plot for subgroup analysis according to the age. The black solid diamond and horizontal lines represent the corresponding SMD and 95% CIs in each study, while the vertical red dotted line suggests the corresponding pooled SMD. The gray boxes point to the weight for each study. The blue diamond corresponds to the overall SMD and 95% CI

**Fig. 5:**
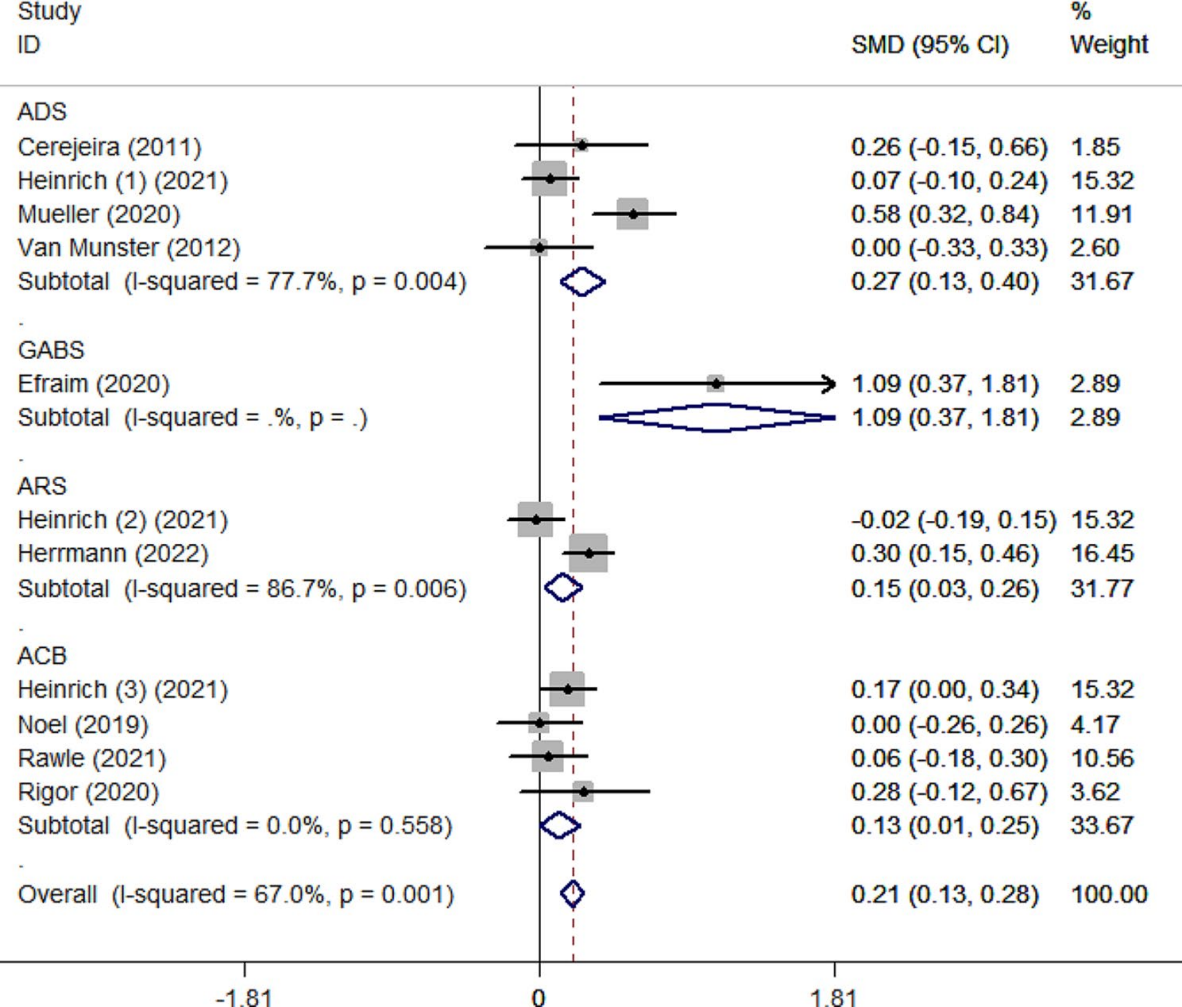
Forest plot for subgroup analysis according to the type of ADB scales. The black solid diamond and horizontal lines represent the corresponding SMD and 95% CIs in each study, while the vertical red dotted line suggests the corresponding pooled SMD. The gray boxes point to the weight for each study. The blue diamond corresponds to the overall SMD and 95% CI

**Fig. 6:**
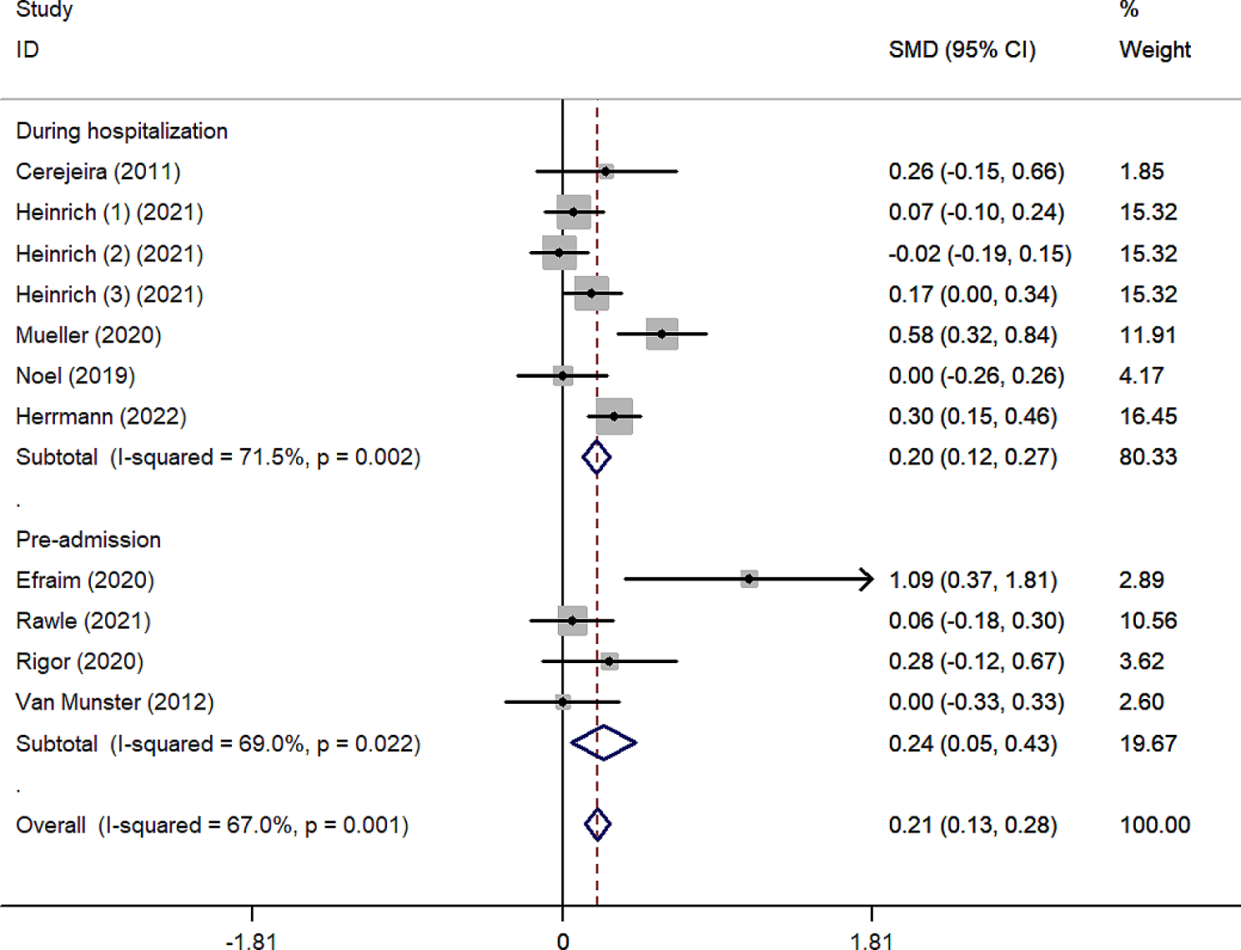
Forest plot for subgroup analysis according to the classification of ADB. The black solid diamond and horizontal lines represent the corresponding SMD and 95% CIs in each study, while the vertical red dotted line suggests the corresponding pooled SMD. The gray boxes point to the weight for each study. The blue diamond corresponds to the overall SMD and 95% CI

As shown in Fig. 2, the pooled SMD for ADB scores between the delirium and non-delirium groups was 0.21 (95%CI 0.13–0.28), indicating that the delirium group had significantly higher ADB scores than the non-delirium group by 0.21 standard deviations. However, due to the small effect size (0.21), the difference within the two groups was slight. The effect size was robust in leave-one-out sensitivity analysis (Supplementary Fig. S1). In addition, the *I²* value was 67%, indicating high heterogeneity between studies (*p* = 0.001). According to the Galbraith plot (Supplementary Fig. S2), the scatter points of all studies were within the parallel line, except for Efraim et al. and Muller et al., indicating that these two studies are relatively heterogeneous compared to the other studies. Subgroup analysis according to admission type (Fig. 3) showed that ADB of patients with delirium was 0.20 and 0.64 standard deviations significantly higher than that of patients without delirium in surgical and internal medicine patients, respectively. This statistical heterogeneity was large in the surgical and internal medicine subgroups (*I*²=70.5%, *p* = 0.002; *I*²=79%, *p* = 0.029), while there was no significant heterogeneity in the emergency subgroup (*I*²=0.00%, *p* = 0.743). In the age subgroups, the SMD was 0.17 (95%CI 0.08–0.26) and 0.27 (95%CI 0.15–0.39) for the age < 75 subgroup and ≥ 75 subgroup, respectively (Fig. 4). The heterogeneity was high in age subgroups (*I²=*71.7%, *p =* 0.003;*I²=*62.7%, *p =* 0.030). We further performed subgroup analysis according to the type of ADB scale, revealing that the GABS subgroup had the largest SMD of 1.09 (95%CI 0.37–1.81), followed by the ADS subgroup (0.27, 95%CI 0.13–0.40), the ARS subgroup (0.15, 95%CI 0.03–0.26) and the ACB subgroup (0.13, 95%CI 0.01–0.25). Except for the ACB subgroup, the heterogeneity among the remaining subgroups was substantial (Fig. 5). The results of the classification of ADB subgroups show that the older people with delirium may have higher pre-admission or during hospitalization ADB scores than those without delirium (Fig. 6).

### Secondary outcome

**Fig. 7:**
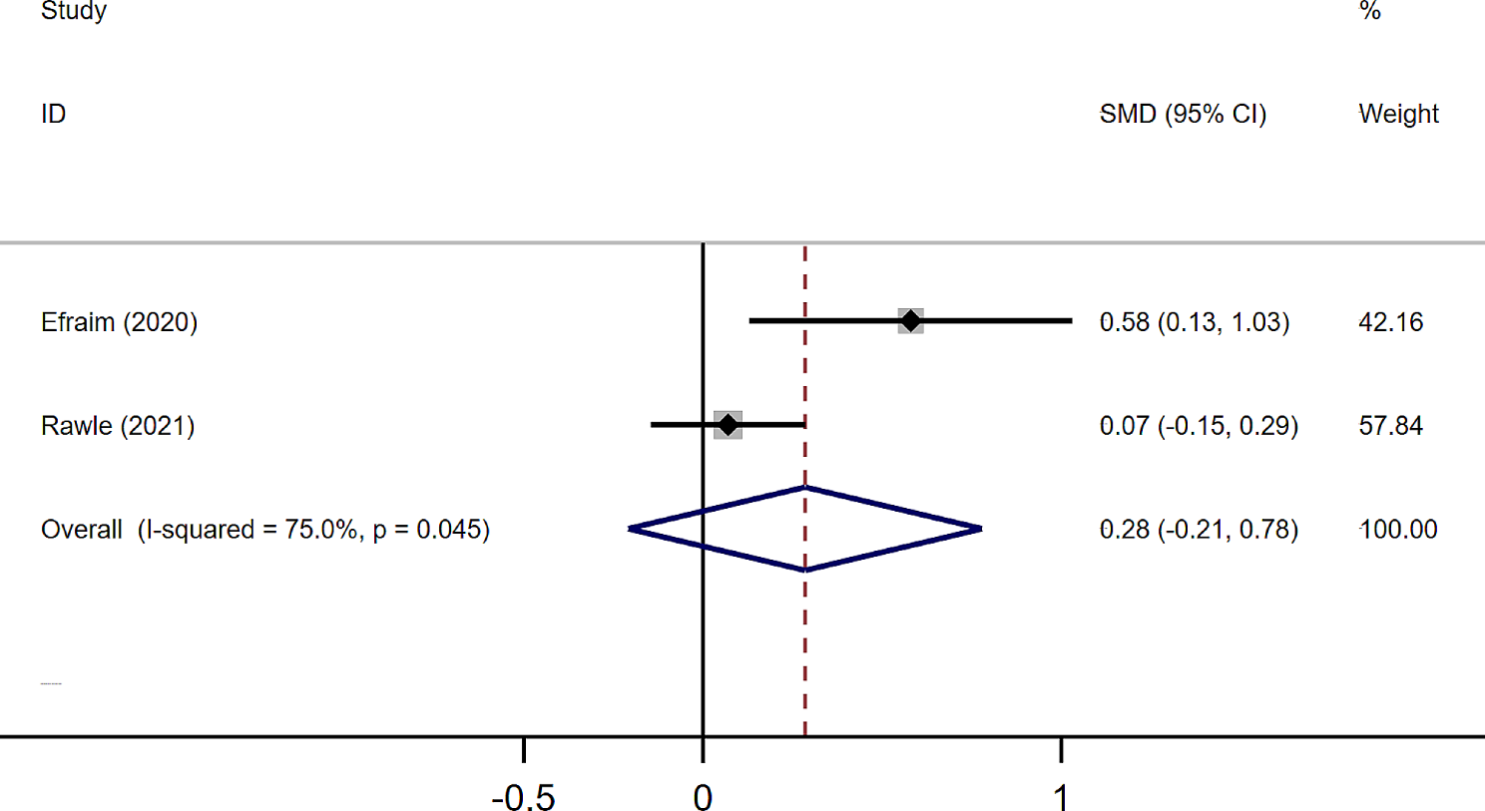
Forest plot for the effects of ADB score on subsyndromal delirium. The black solid diamond and horizontal lines represent the corresponding SMD and 95% CIs in each study, while the vertical red dotted line suggests the corresponding pooled SMD. The gray boxes point to the weight for each study. The blue diamond corresponds to the overall SMD and 95% CI

Two of the nine studies further explored the effect of ADB on subsyndromal delirium. For example, in the study by Efraim et al. delirium was assessed by CAM [17]. Delirium was diagnosed when both core criteria (acute onset and fluctuating course, and inattention) and at least one other criteria (altered level of attention or disorganized thinking) were met [25]. In contrast, subsyndromal delirium satisfies one of the core criteria and at least one other criteria. As shown in Fig. 7, the pooled SMD for the subsyndromal delirium group versus the non-delirium group was 0.29 (95%CI -0.02-0.37), with high heterogeneity (*I²*=75.0%, *p* = 0.045).

### Publication bias

The funnel plot (Supplementary Fig. S3) presents a symmetric distribution of all studies, with only two studies outside the funnel. Meanwhile, Egger’s test revealed no significant publication bias (*t* = 1.10, *p* = 0.300).

## Discussion

This systematic review and meta-analysis aimed to investigate the quantitative relationship between ADB and the occurrence of delirium in older hospitalized patients. Our findings revealed a significant difference in ADB between delirious and non-delirious older patients, with a higher cumulative anticholinergic activity observed in delirious patients. Differences in ADB were also evident in patients admitted for surgery and internal medicine treatment. The effect of ADB on delirium was also observed in age, ADB scale type and classification of ADB subgroups. However, no differences were found between patients with subsyndromal delirium and those without delirium.

Several previous systematic reviews have investigated the relationship between anticholinergic drug use and the development of delirium [26–28]. In a review by Salahudeen et al., total serum anticholinergic activity (SAA) after administration of anticholinergic drugs was associated with an increased risk of delirium [26]. However, the total SAA includes both endogenous and exogenous anticholinergic activity, making it difficult to distinguish the exogenous anticholinergic effect of drugs. Additionally, the definition of ADB is unclear, making it difficult to compare the results as most original studies in those review didn’t use the ADB scale to quantify ADB [27, 28]. Several systematic reviews have compared the associations between different ADB scales and delirium, with the aim of finding the most relevant ADB scale to identify people at high risk of delirium [8, 29]. Overall, the studies included in these systematic reviews were heterogeneous, mainly due to differences in study population and design. They only reported the effect size of each study and did not perform meta-analysis to provide a quantitative relationship between ADB and delirium in older hospitalized patients. In this review, we selected nine high-quality prospective cohort studies for systematic review and meta-analysis, resulting in low heterogeneity among the included studies.

Our findings indicate that delirious older patients had significantly higher ADB compared to non-delirious patients, although the small effect size (SMD = 0.21) suggests a minor difference between them. In addition, we hypothesized that admission type and age were important factors affecting the outcome, as different admission types may reflect different pathophysiological states, resulting in different sensitivity to anticholinergic medications. Subgroup analysis revealed that the SMD in the internal medicine subgroup reached a moderate effect size (SMD = 0.64), which is higher than the overall SMD (0.21), indicating that ADB may have a stronger effect on delirium in older patients admitted for internal medicine. This may be attributed to drug treatment being the primary approach in internal medicine departments, particularly the use of anticholinergic medications, leading to more anticholinergic-related delirium in this group. ADB may influence delirium in surgical older patients as well (SMD = 0.2), which was close to the overall SMD. Anticholinergics are important preoperative medications for ensuring clinical anesthesia safety but also pose risks for postoperative delirium [30]. Regarding age, we selected 75 years as the threshold for dividing the age subgroups based on the median age of patients from the nine studies. In both the < 75 and ≥ 75 years subgroup, delirious patients had significantly higher ADB scores than non-delirious patients (similar to the overall SMD). Central cholinergic neurons gradually decline with age, leading to central cholinergic defects. Older patients are more susceptible to adverse reactions associated with anticholinergic medications due to age-related pharmacokinetic and pharmacodynamic changes [5]. The comparison of SMD between the < 75 and ≥ 75 years subgroup suggests that the effect of ADB on delirium may strengthen with increasing age. The ADS scale was used most frequently among the nine included studies, followed by the ACB, ARS, and GABS scales. The impact of ADB on delirium can be observed regardless of the different scale used. However, the strength of the effect may depend on the type of scale. The GABS subgroup may show a strong effect size (1.09), while the SMD in the other scale subgroups were relatively close, representing small effect sizes. It is important to note that the GABS subgroup had only one article, and further validation is needed. The results of the ADB classification subgroup indicated that either long-term accumulation of ADB before admission or short-term use of anticholinergic drugs during hospitalization may play an important role in delirium of older people.

In the overall analysis and most subgroup analyses, it was observed that the actual difference in ADB between delirious and non-delirious patients may be relatively small. Except the limited studies in each subgroup, the property of anticholinergic burden may be the potential reason. The studies calculated pre-admission ADB did not take into account ADB during hospitalization. The ADB scores were low in both the delirium and non-delirium groups (Table 1), suggesting that the use of anticholinergic drugs, especially potent anticholinergic drugs, may be less frequent before admission. Rigor et al. found that the proportion of inpatient prescriptions for anticholinergic drugs was significantly higher than outpatient prescriptions (93.4% vs. 72.7%), and the proportion of potent anticholinergic drugs (ACB score ≥ 3 points) was twice as high as outpatient prescriptions (53% vs. 27.3%) [12]. According to Beers and *STOPP*/START criteria, potent anticholinergic drugs are an independent risk factor for delirium [6, 31]. Patients with delirium used more potent anticholinergic drugs during hospitalization than those without delirium [32]. Moreover, the total ADB scores in the nine studies were obtained by summing the scores of each drug without adjustment for dose and duration of therapy, which may not accurately reflect the ADB in vivo [33]. Even for the same total ADB score, different score compositions could affect the results. For example, Hsu et al. found that a total ACB score consisting of several low-scoring drugs was associated with emergency department visits and all-cause hospitalizations, whereas a single high-scoring drug was associated with the risk of fracture admission and dementia [33].

Subsyndromal delirium is a transitional state between normal mental status and delirium, with a prevalence of 36.4% in the older people [34]. Early identification and intervention of subsyndromal delirium can reduce the risk of developing delirium. The SMD for ADB between the subsyndromal delirium and non-delirium groups was 0.29 (95%CI -0.21 to 0.78). We did not observe an impact of ADB on subsyndromal delirium. However, due to the small number of studies (only two) and high heterogeneity (*I*^2^ = 75%, *p* = 0.045), more studies are needed to confirm this finding.

This systematic review and meta-analysis have several advantages. First, we used SMD as the pooled effect size. Since different ADB scales were used in each study to calculate ADB, the SMD could eliminate the effect of absolute magnitude and the effect of the metric on the outcome [35]. Second, we performed multiple subgroup analyses based on factors that might contribute to study heterogeneity (e.g., admission type, age, and ADB scales). Additionally, we investigated the impact of ADB on subsyndromal delirium, which is clinically neglected but has a high prevalence among older patients. The greatest limitation of this review is that few studies were included despite the high quality of the studies. There are no randomized clinical trials (RCTs) investigating whether higher ADB increases the risk of delirium in older patients. Therefore, we selected prospective cohort studies of relatively high evidence-based quality for the meta-analysis. Third, some subgroups were highly heterogeneous. In addition to the small number of studies, there may be unnoticed factors that were not classified in our review that may play an important role in the effect of ADB on delirium.

## Conclusions

We conducted a comprehensive systemic review and meta-analysis of 3791 patients from nine prospective cohort studies to establish the impact of ADB on delirium in older hospitalized patients. Our findings revealed that ADB was significantly higher in delirious patients than in non-delirious patients, with this difference remaining significant across different age, ADB scale type and the ADB classification subgroups. However, the effect of ADB on delirium is only observed in specific older inpatients. Currently, most studies focus on older patients in emergency, surgery, and internal medicine in Europe and America. In the future, it is crucial to conduct more studies outside of Europe and America to explore the correlation between ADB levels and delirium among critically ill older patients.

### Electronic supplementary material

Below is the link to the electronic supplementary material.

Supplementary Material 1

## Data Availability

Data is provided within the manuscript or supplementary information files.
