# Supplementary material for: Differences of anticholinergic drug burden between older hospitalized patients with and without delirium: a systematic review and meta-analysis based on prospective cohort studies
Source: BMC Geriatr. 2024 Jul 12;24:599. doi: 10.1186/s12877-024-05197-6 (PMC11241997; doi:10.1186/s12877-024-05197-6)
Supplement: Supplementary file 1 — Supplementary Material 1 [file 12877_2024_5197_MOESM1_ESM.docx]

**Supplementary Table S1.** PRISMA Checklist

| **Section and Topic** | **Item #** | **Checklist item** | **Location where item is reported** |
| --- | --- | --- | --- |
| **TITLE** | | |  |
| Title | 1 | Identify the report as a systematic review. | Title page |
| **ABSTRACT** | | |  |
| Structured summary | 2 | Provide a structured summary including, as applicable: background; objectives; data sources; study eligibility criteria, participants, and interventions; study appraisal and synthesis methods; results; limitations; conclusions and implications of key findings; systematic review registration number. | 1 |
| **INTRODUCTION** | | |  |
| Rationale | 3 | Describe the rationale for the review in the context of existing knowledge. | 3 |
| Objectives | 4 | Provide an explicit statement of the objective(s) or question(s) the review addresses. | 3 |
| **METHODS** | | |  |
| Eligibility criteria | 5 | Specify the inclusion and exclusion criteria for the review and how studies were grouped for the syntheses. | 4 |
| Information sources | 6 | Specify all databases, registers, websites, organisations, reference lists and other sources searched or consulted to identify studies. Specify the date when each source was last searched or consulted. | 4 |
| Search strategy | 7 | Present the full search strategies for all databases, registers and websites, including any filters and limits used. | Supp. Table S2 |
| Selection process | 8 | Specify the methods used to decide whether a study met the inclusion criteria of the review, including how many reviewers screened each record and each report retrieved, whether they worked independently, and if applicable, details of automation tools used in the process. | 5 |
| Data collection process | 9 | Specify the methods used to collect data from reports, including how many reviewers collected data from each report, whether they worked independently, any processes for obtaining or confirming data from study investigators, and if applicable, details of automation tools used in the process. | 5 |
| Data items | 10a | List and define all outcomes for which data were sought. Specify whether all results that were compatible with each outcome domain in each study were sought (e.g. for all measures, time points, analyses), and if not, the methods used to decide which results to collect. | 5 |
|  | 10b | List and define all other variables for which data were sought (e.g. participant and intervention characteristics, funding sources). Describe any assumptions made about any missing or unclear information. | Table 1 |
| Study risk of bias assessment | 11 | Specify the methods used to assess risk of bias in the included studies, including details of the tool(s) used, how many reviewers assessed each study and whether they worked independently, and if applicable, details of automation tools used in the process. | 6 |
| Effect measures | 12 | Specify for each outcome the effect measure(s) (e.g. risk ratio, mean difference) used in the synthesis or presentation of results. | 6 |
| Synthesis methods | 13a | Describe the processes used to decide which studies were eligible for each synthesis (e.g. tabulating the study intervention characteristics and comparing against the planned groups for each synthesis (item #5)). | 6 |
|  | 13b | Describe any methods required to prepare the data for presentation or synthesis, such as handling of missing summary statistics, or data conversions. | 6 |
|  | 13c | Describe any methods used to tabulate or visually display results of individual studies and syntheses. | 6 |
|  | 13d | Describe any methods used to synthesize results and provide a rationale for the choice(s). If meta-analysis was performed, describe the model(s), method(s) to identify the presence and extent of statistical heterogeneity, and software package(s) used. | 6 |
|  | 13e | Describe any methods used to explore possible causes of heterogeneity among study results (e.g. subgroup analysis, meta-regression). | 6 |
|  | 13f | Describe any sensitivity analyses conducted to assess robustness of the synthesized results. | 7 |
| Reporting bias assessment | 14 | Describe any methods used to assess risk of bias due to missing results in a synthesis (arising from reporting biases). | 6 |
| Certainty assessment | 15 | Describe any methods used to assess certainty (or confidence) in the body of evidence for an outcome. | 7 |
| **RESULTS** | | |  |
| Study selection | 16a | Describe the results of the search and selection process, from the number of records identified in the search to the number of studies included in the review, ideally using a flow diagram. | Fig. 1 |
|  | 16b | Cite studies that might appear to meet the inclusion criteria, but which were excluded, and explain why they were excluded. | Fig. 1 |
| Study characteristics | 17 | Cite each included study and present its characteristics. | Table 1 |
| Risk of bias in studies | 18 | Present assessments of risk of bias for each included study. | Supp. Table S3 |
| Results of individual studies | 19 | For all outcomes, present, for each study: (a) summary statistics for each group (where appropriate) and (b) an effect estimate and its precision (e.g. confidence/credible interval), ideally using structured tables or plots. | Table 1 |
| Results of syntheses | 20a | For each synthesis, briefly summarise the characteristics and risk of bias among contributing studies. | 8 |
|  | 20b | Present results of all statistical syntheses conducted. If meta-analysis was done, present for each the summary estimate and its precision (e.g. confidence/credible interval) and measures of statistical heterogeneity. If comparing groups, describe the direction of the effect. | 9 |
|  | 20c | Present results of all investigations of possible causes of heterogeneity among study results. | 9 |
|  | 20d | Present results of all sensitivity analyses conducted to assess the robustness of the synthesized results. | 9 |
| Reporting biases | 21 | Present assessments of risk of bias due to missing results (arising from reporting biases) for each synthesis assessed. | 10 |
| Certainty of evidence | 22 | Present assessments of certainty (or confidence) in the body of evidence for each outcome assessed. | 9 |
| **DISCUSSION** | | |  |
| Discussion | 23a | Provide a general interpretation of the results in the context of other evidence. | 10-11 |
|  | 23b | Discuss any limitations of the evidence included in the review. | 11-12 |
|  | 23c | Discuss any limitations of the review processes used. | 13 |
|  | 23d | Discuss implications of the results for practice, policy, and future research. | 14 |

**Supplementary Table S2.** search strategy

| Database | Search strategy |
| --- | --- |
| PubMed | ((((Aged[MeSH Terms]) OR (Elderly[Title/Abstract])) OR (Geriatric[Title/Abstract])) AND ((((((((((Cholinergic Antagonist*[MeSH Terms]) OR (Cholinergic Receptor Antagonist*[Title/Abstract])) OR (Cholinergic-Blocking Agent*[Title/Abstract])) OR (Cholinergic Blocking Agent*[Title/Abstract])) OR (Cholinolytic*[Title/Abstract])) OR (Acetylcholine Antagonist*[Title/Abstract])) OR (Anticholinergic Agent*[Title/Abstract])) OR (Anti-Cholinergic*[Title/Abstract])) OR (Anti Cholinergic*[Title/Abstract])) OR (Anticholinergic*[Title/Abstract]))) AND ((((Delirium[MeSH Terms]) OR (Subacute Delirium*[Title/Abstract])) OR (Delirium of Mixed Origin[Title/Abstract])) OR (Mixed Origin Delirium*[Title/Abstract])) |
| Embase | ('aged'/exp OR 'elderly':ab,ti OR 'geriatric':ab,ti) AND ('cholinergic receptor blocking agent'/exp OR 'cholinergic receptor antagonist*':ab,ti OR 'cholinergic-blocking agent*':ab,ti OR 'cholinergic blocking agent*':ab,ti OR 'cholinolytic*':ab,ti OR 'acetylcholine antagonist*':ab,ti OR 'anticholinergic agent*':ab,ti OR 'anti-cholinergic*':ab,ti OR 'anti cholinergic*':ab,ti OR 'anticholinergic*':ab,ti) AND ('delirium'/exp OR 'subacute delirium*':ab,ti OR 'delirium of mixed origin':ab,ti OR 'mixed origin delirium*':ab,ti) AND [humans]/lim |
| Web of Science | TS= (Aged OR Elderly OR Geriatric) AND (Cholinergic Antagonist* OR Cholinergic-Blocking Agent* OR Cholinergic Receptor Antagonist* OR Cholinergic Blocking Agent* OR Cholinolytic* OR Acetylcholine Antagonist* OR Anticholinergic Agent* OR Anti-Cholinergic* OR Anti Cholinergic* OR Anticholinergic*) AND (Delirium OR Subacute Delirium* OR Delirium of Mixed Origin OR Mixed Origin Delirium*) |
| Cochrane Library | (MeSH descriptor: [Aged] OR (Elderly):ti,ab,kw OR (Geriatric):ti,ab,kw) AND (MeSH descriptor: [Cholinergic Antagonists] OR (Cholinergic Receptor Antagonist*):ti,ab,kw OR (Cholinergic-Blocking Agent*):ti,ab,kw OR (Cholinergic Blocking Agent*):ti,ab,kw OR (Cholinolytic*):ti,ab,kw OR (Acetylcholine Antagonist*):ti,ab,kw OR (Anticholinergic Agent*):ti,ab,kw OR (Anti-Cholinergic*):ti,ab,kw OR (Anti Cholinergic*):ti,ab,kw OR (Anticholinergic*):ti,ab,kw) AND (MeSH descriptor: [Delirium] OR (Subacute Delirium*):ti,ab,kw OR (Delirium of Mixed Origin):ti,ab,kw OR (Mixed Origin Delirium*):ti,ab,kw) |
| CINAHL EBSCOhost | (MH Aged OR TI (Elderly OR Geriatric) OR AB (Elderly OR Geriatric)) AND (MH Cholinergic Antagonist* OR TI (Cholinergic Receptor Antagonist* OR Cholinergic-Blocking Agent* OR Cholinergic Blocking Agent* OR Cholinolytic* OR Acetylcholine Antagonist* OR Anticholinergic Agent* OR Anti-Cholinergic* OR Anti Cholinergic* OR Anticholinergic*) OR AB (Cholinergic Receptor Antagonist* OR Cholinergic-Blocking Agent* OR Cholinergic Blocking Agent* OR Cholinolytic* OR Acetylcholine Antagonist* OR Anticholinergic Agent* OR Anti-Cholinergic* OR Anti Cholinergic* OR Anticholinergic*)) AND (MH Delirium OR TI (Subacute Delirium* OR Delirium of Mixed Origin OR Mixed Origin Delirium*) OR AB (Subacute Delirium* OR Delirium of Mixed Origin OR Mixed Origin Delirium*)) |

# **Supplementary Table S3.** Study Quality Assessment (Newcastle-Ottawa Scale)

| Study | Year | Selection | | | | Comparability | Outcome | | | Total |
| --- | --- | --- | --- | --- | --- | --- | --- | --- | --- | --- |
|  |  | Representative of Exposed Cohort | Selection of Nonexposed Cohort | Ascertainment of Exposure | Demonstration - Outcome not Present at start | Adjustment | Ascertainment of Outcome | Was Follow up long Enough | Adequacy of Follow up of Cohorts |  |
| Cerejeira | 2011 | 1 | 1 | 1 | 1 | 1 | 1 | 1 | 1 | 8 |
| Efraim | 2020 | 1 | 1 | 1 | 1 | 2 | 1 | 1 | 1 | 9 |
| Heinrich | 2021 | 1 | 1 | 1 | 1 | 2 | 1 | 1 | 1 | 9 |
| Muller | 2020 | 1 | 1 | 1 | 1 | 2 | 1 | 1 | 1 | 9 |
| Noel | 2019 | 1 | 1 | 1 | 1 | 2 | 1 | 1 | 1 | 9 |
| Rawle | 2021 | 1 | 1 | 1 | 1 | 0 | 1 | 1 | 1 | 7 |
| Rigor | 2020 | 1 | 1 | 1 | 0 | 2 | 1 | 1 | 1 | 8 |
| Van Munster | 2012 | 1 | 1 | 1 | 1 | 1 | 1 | 1 | 1 | 8 |
| Herrmann | 2022 | 1 | 1 | 1 | 0 | 2 | 1 | 1 | 1 | 8 |

***Note:*** The total score ranges from 7 to 9 represents high quality, 4-6 represents high risk, and 0-3 represents very high risk of bias


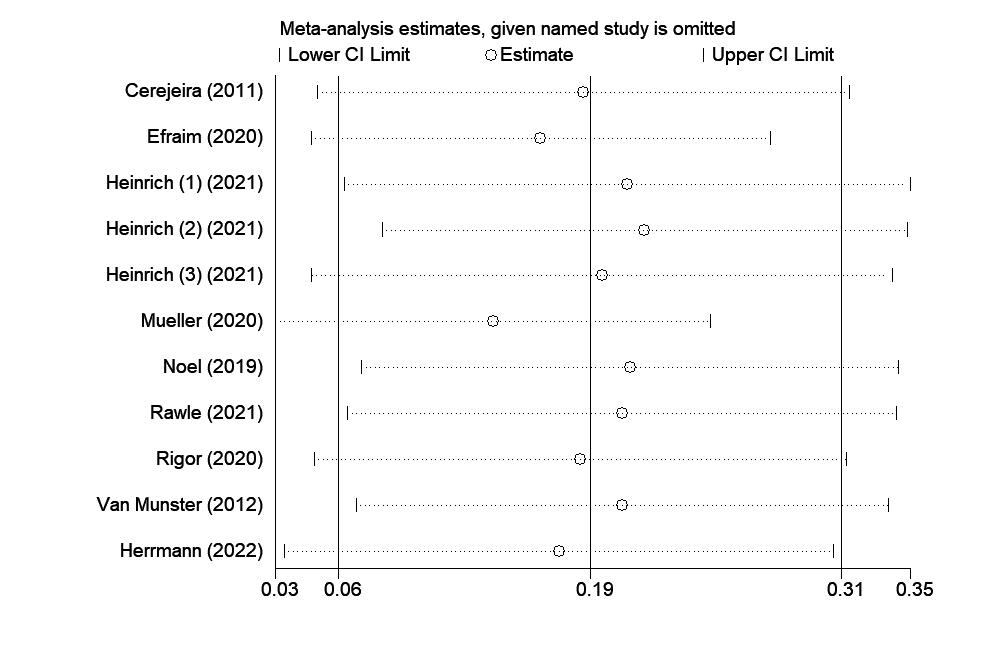


**Supplementary Fig. S1.** leave-one-out sensitivity analysis. The three solid vertical lines denote the pooled fixed effect of OR and 95% CI (OR = 0.14, 95% CI 0.03-0.24). The horizontal lines and the circles indicate the ORs and 95% CIs applying the leave-one-out method.


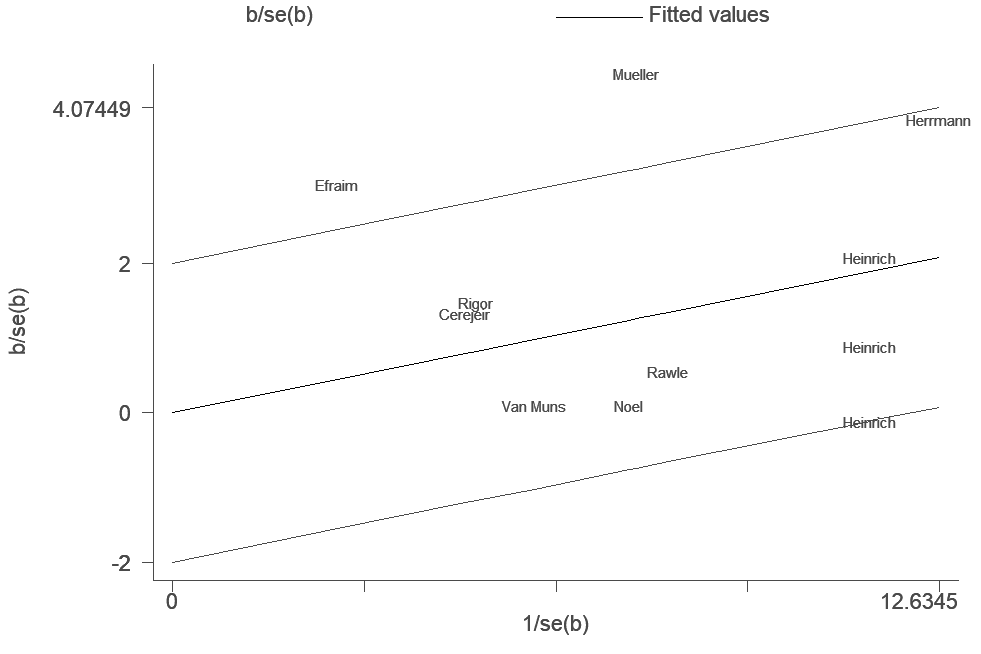


**Supplementary Fig. S2.** Galbraith plot for heterogeneity test.


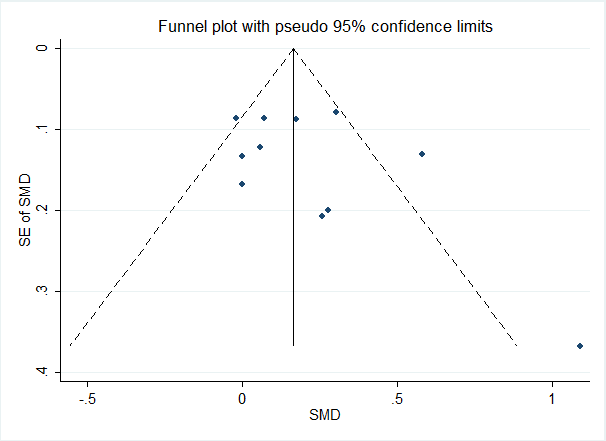


**Supplementary Fig. S3.** funnel plot of standard difference, with SMD on the X-axis and standard error on the Y-axis for the effect of ADB scores on delirium.
